# Supplementary material for: Cancer cells surviving cisplatin chemotherapy increase stress-induced OMA1 activity and mitochondrial fragmentation
Source: bioRxiv. 2025 Oct 1:2025.09.29.679325. Preprint. [Version 1] doi: 10.1101/2025.09.29.679325 (PMC12622012; doi:10.1101/2025.09.29.679325)
Supplement: Supplement 1 — Supplementary Figure S1. N-acetyl cysteine decreases levels of reactive oxygen species in cells 10 Days Post-Treatment Removal. (a) Representative DCF-DA fluorescence images of cells treated with N-acetyl cysteine and vehicle. (b) Quantification of mean fluorescence intensity of DCF-DA between indicated groups. Supplementary Figure S2. Uncropped blot images from Figure 2a. Boxed areas delineate the cropped portion displayed in Figure 2a. Supplementary Figure S3. Uncropped blot images from Figure 3a. Boxed areas delineate the cropped portion displayed in Figure 3a. Supplementary Video 1. Representative time lapse of mitochondrial dynamics in untreated PC3 cells stained with MitoTracker Green. Supplementary Video 2. Second representative time lapse of mitochondrial dynamics in untreated PC3 cells stained with MitoTracker Green. Supplementary Video 3. Representative time lapse of mitochondrial dynamics in cells 10 Days Post-Treatment Removal stained with MitoTracker Green. Supplementary Video 4. Second representative time lapse of mitochondrial dynamics in cells 10 Days Post-Treatment Removal stained with MitoTracker Green. [file media-1.pdf]

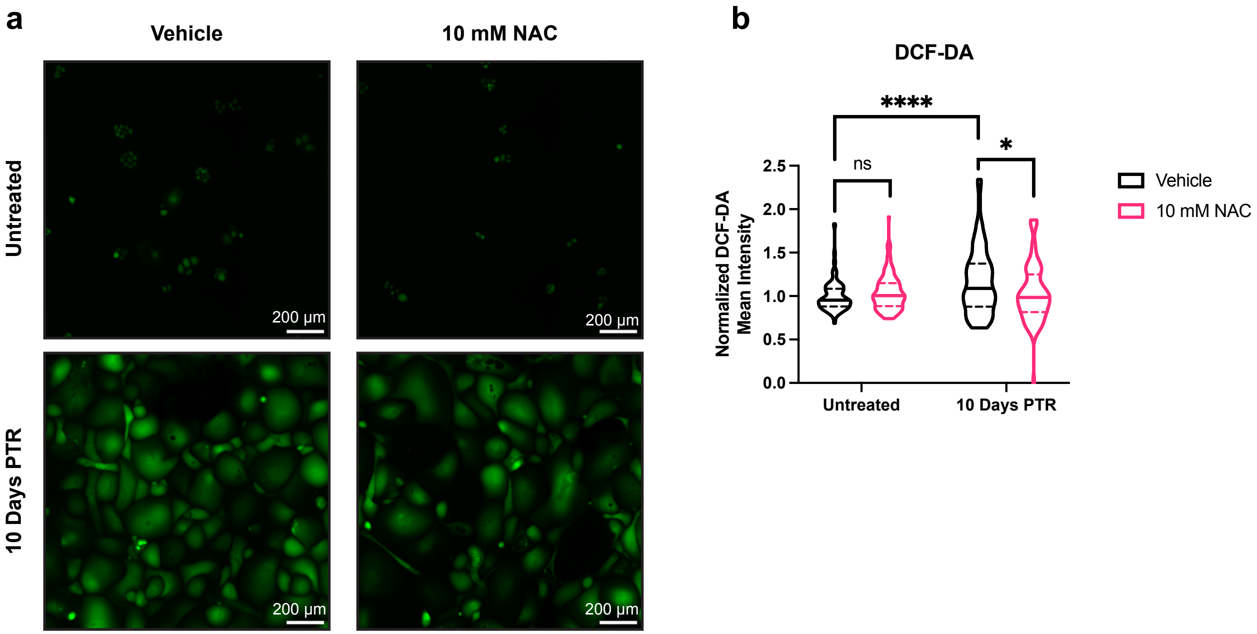

Supplementary Figure S1. N-acetyl cysteine decreases levels of reactive oxygen species in cells 10 Days Post-Treatment Removal. (**a**) Representative DCF-DA fluorescence images of cells treated with N-acetyl cysteine and vehicle. (**b**) Quantification of mean fluorescence intensity of DCF-DA between indicated groups.

Supplementary Figure S2. Uncropped blot images from Figure 2a. Boxed areas delineate the cropped portion displayed in Figure 2a.

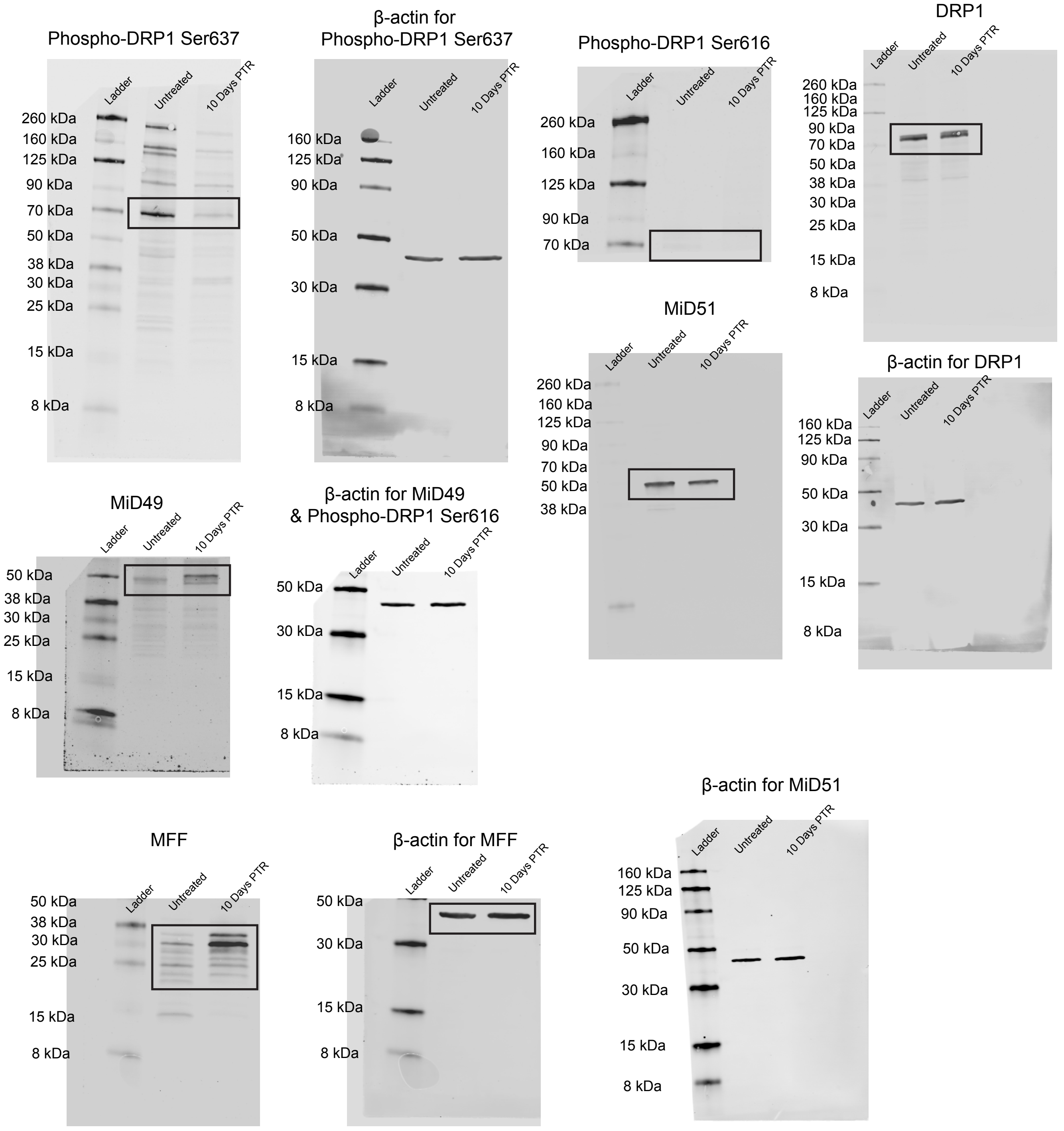

Supplementary Figure S3. Uncropped blot images from Figure 3a. Boxed areas delineate the cropped portion displayed in Figure 3a.

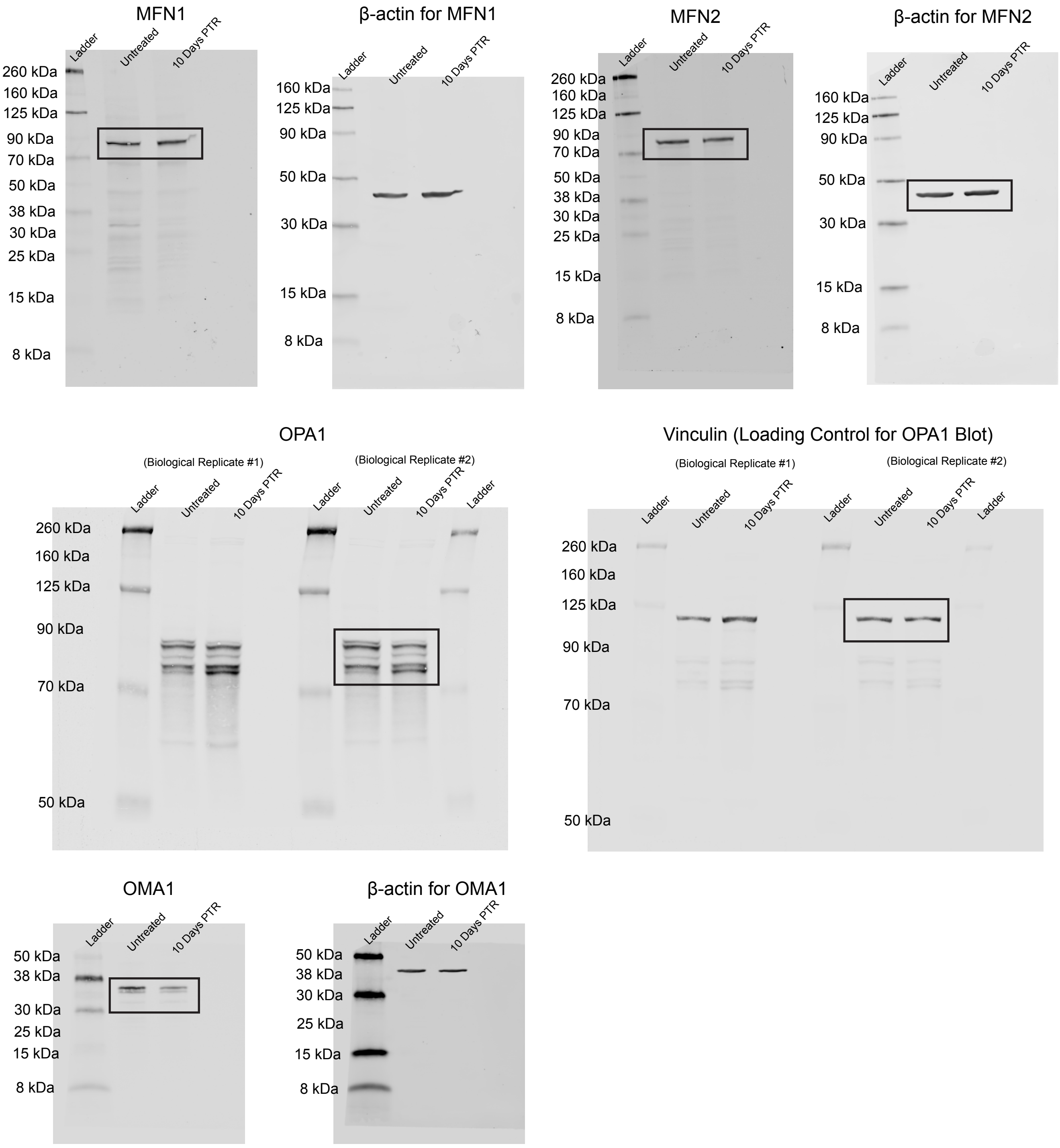

Supplementary Videos 1-4.

Due to the video size exceeding bioRxiv's 40 MB limit, the supplementary videos are deposited in a OneDrive Folder and can be viewed through the link below.

https://livejohnshopkins-my.sharepoint.com/:f:/g/personal/mli154_jh_edu/EkQbnbLWvZhKu1v6M18h5VoBo30PW1Ie-EjUgUkBh7vBtw
